# Electrospun Polyvinylpyrrolidone-Gelatin and Cellulose Acetate Bi-Layer Scaffold Loaded with Gentamicin as Possible Wound Dressing

**DOI:** 10.3390/polym12102311

**Published:** 2020-10-09

**Authors:** Héctor D. López-Calderón, Hamlet Avilés-Arnaut, Luis J. Galán-Wong, Verónica Almaguer-Cantú, J. R. Laguna-Camacho, C. Calderón-Ramón, J. E. Escalante-Martínez, Katiushka Arévalo-Niño

**Affiliations:** 1 Facultad de Ciencias Biológicas, Instituto de Biotecnología, Universidad Autónoma de Nuevo León, UANL, San Nicolás de los Garza, Nuevo León 66455, Mexico; hector.lopezcl@uanl.edu.mx (H.D.L.-C.); hamlet.avilesarn@uanl.edu.mx (H.A.-A.); luis.galanwn@uanl.edu.mx (L.J.G.-W.); veronica.almaguerct@uanl.edu.mx (V.A.-C.); 2 Facultad de Ingeniería Mecánica y Eléctrica., Universidad Veracruzana, Av. Venustiano Carranza s/n, col. Revolución, Poza Rica, Veracruz 93390, Mexico; jlaguna@uv.mx (J.R.L.-C.); ccalderon@uv.mx (C.C.-R.); jeescalante@uv.mx (J.E.E.-M.)

**Keywords:** electrospinning, biomaterial, nanofibers, wound dressing, antibacterial

## Abstract

Acceleration of wound healing can be achieved with the use of wound dressings. Through the electrospinning technique, a polymeric scaffold composed of two layers was processed: a gelatin and polyvinylpyrrolidone layer with gentamicin, and a second layer of cellulose acetate. The conditions for the electrospinning process were standardized for voltage parameters, feed flow and the distance from the injector to the collector. Once the values of the main variables for the electrospinning were optimized, a three-hour processing time was established to allow the separation of the material from the collector. The obtained material was characterized by observations on scanning electron microscopy, Fourier transform infrared spectroscopy and thermal analysis; contact angle measurement was performed to evaluate wettability properties, and antibacterial activity against *Pseudomonas aeruginosa* and *Staphylococcus aureus* were evaluated using the Kirby–Bauer test. The obtained fibers that form the bi-layer scaffold present diameters from 100 to 300 nm. The scaffold presents chemical composition, thermal stability, wettability characteristics and antibacterial activity that fulfill the proposal from this study, based on obtaining a scaffold that could be used as a drug delivery vehicle and a wound dressing material.

## 1. Introduction

The development of scaffolds suitable for the regeneration of damaged tissues with full recovery of their biological functions has long been an important objective in tissue engineering, and electrospinning is one of the processes that has gained great importance as a proposal for the development of such scaffolds. Due to their fibrous structure, electrospun materials can serve as a scaffold for the skin cells in the auto-repairing process, and by being permeable to moisture and air, allows for the adequate extraction of extra body fluid from the wound area to avoid infection and maintain a moisturized environment [1,2]. This moisturized environment could reduce the time for wound healing and the formation of scars, since in a wet environment skin is regenerated without the formation of a scab [3]. In addition to the previously mentioned characteristics, antibacterial agents can be loaded to the electrospun scaffolds, thus obtaining active materials that could fulfill functions as wound dressings [4,5]. Bacterial infections are considered to be a major complication for skin wounds due to the stimulation of inflammatory cells to secrete pro-inflammatory cytokines, inducing exudate formation, delay wound healing, and facilitate improper collagen deposition [1]. Polyvinyl pyrrolidone (PVP) is an important polymer, with properties such as good adhesion, low toxicity, high hygroscopicity, excellent physiological compatibility and high solubility in water and organic solvents, with a long history in biomedical and pharmaceutical applications [6]. Gelatin (GEL) is obtained from denaturing the triple helical structure of collagen [7]. This biopolymer has as many as 20 different amino acids in variable proportions in its composition, providing RGD (l-arginine-glycine-l-aspartic acid), a three-amino-acid recognition sequence for integrin mediated cell adhesion. GEL also exhibits good biocompatibility, biodegradability and non-immunogenic characteristics; hence, it finds numerous applications in the food and pharmaceutical industries [8]. Unlike collagen, GEL does not induce antigenicity, a behavior associated with the absence or low presence of aromatic amino acids like tyrosine, tryptophan and phenylalanine in the latter, which results in a significantly lower amount of immunogenic aromatic radicals than among the former polymer [9]. Due to disadvantages such as low mechanical resistance, high viscosity, rapid enzymatic degradation and low solubility, GEL is generally combined with other polymers to improve its mechanical properties and promote the biocompatibility of these polymers [10]; in this case, a mixture of GEL with PVP was made.

Cellulose acetate (CA) is one of the most important cellulose derivatives [11]. In recent decades, great attention has been paid to fibers from cellulose and its derivatives due to their low cost, light weight, easy processing, good mechanical and barrier properties and recyclability [12]. It demonstrates great potential in medical material and tissue engineering applications as a functional fiber, due to its superior properties such as biodegradability, thermal stability, biocompatibility and non-toxicity [13,14]. Moreover, CA adequately fulfills a role as structural reinforcement through interactions between polymers and cellulosic materials through hydrogen bonds with amino groups, improving the mechanical properties of the compounds [15,16]. Therefore, it was decided to include CA in the formulations for the development of this work.

Gentamicin (GEN) is an aminoglycoside commonly used in the treatment and prophylaxis of bacterial infections. Similarly to other aminoglycosides, it has a relatively short half-life, low bioavailability, and may cause side effects such as ototoxicity and nephrotoxicity [17,18].

In this study, through the electrospinning technique, a polymeric scaffold composed of two layers a gelatin and polyvinylpyrrolidone layer with gentamicin and a second layer of cellulose acetate was processed; the scaffold was characterized, and the antibacterial activity against *Pseudomonas aeruginosa* and *Staphylococcus aureus* was evaluated.

## 2. Materials and Methods

### 2.1. Materials

PVP (*M*_w_ 360,000), GEL (gel strength 300, Type A) and CA (*M*_n_ 30,000; acetyl groups ∼40%) were purchased from Sigma-Aldrich (St. Louis, MO, USA). The acetic acid (ACS reagent grade) and absolute ethanol (ACS reagent grade) used as solvents were purchased from Probiotek (Monterrey, Mexico). The gentamicin (80 mg·mL^−1^) used was purchased from Comercializadora Farmacéutica de Chiapas (Monterrey, Mexico).

### 2.2. Polymeric Solutions

Polymer solutions of GEL and PVP (16% *w*/*v*) were prepared using acetic acid (20% *v*/*v*) and ethanol, as solvents, respectively. In summary, for the preparation of the solutions, magnetic stirring plates were used at room temperature overnight. After this period, the solutions were mixed in a polyvinylpyrrolidone:gelatin (PG) ratio of 1:4, continuing with agitation until a homogeneous solution was obtained. The amount of gentamicin necessary to obtain a concentration of 3% (*v*/*v*) (PG+gen) was added to the solution. The CA solution was prepared at 16% (*w*/*v*) in the same way as the other two solutions, using glacial acetic acid as the solvent. The whole solutions preparation processes were performed at room temperature (23 ± 2 °C).

### 2.3. Electrospinning

Once the solutions were obtained, they were processed by electrospinning using a Standard Unit NEU-01 (Tong Li Tech Co, Nanshan, Shenzhen, China). The conditions for the elaboration of the fibers, formed by the PG+gen solution, were 0.2 mL·h^−1^ of feed flow of the solution, 20 kV of electrical potential difference and 15 cm of distance between the injector and the collector. Once the PG+gen layer was formed, the CA solution was processed so that this layer was collected on top of the former to obtain the scaffold. The conditions for processing the CA solution used were 0.3 mL·h^−1^ of solution feed flow, 26 kV electrical potential difference and 15 cm distance between injector and collector. All repetitions of the process were carried out at 25.0 ± 0.5 °C.

### 2.4. Characterization of the Scaffold

#### 2.4.1. Scanning Electron Microscopy—SEM

Characteristics of morphology and topography were analyzed by Scanning Electron Microscopy (SEM) (Nova NanoSEM 200, FEI, Thermo Fisher Scientific, Waltham, MA, US) of the fibers obtained. The samples were coated with gold for 180 s prior visualization. The average diameter of the fibers was determined using the ImageJ v1.52a program [19]. To obtain the average diameter of the fibers and pores, 100 different measurements of each were taken within the samples.

#### 2.4.2. Attenuated Total Reflection Fourier Transform Infrared Spectroscopy—ATR-FTIR

Detection of the signals corresponding to the characteristic chemical groups of the materials used to produce the polymeric scaffold was analyzed by attenuated total reflection Fourier transform infrared spectroscopy (ATR/FTIR), carried out with a Nicolet iS10 equipped with a Smart OMNI-Transmission accessory (Thermo Scientific, Waltham, MA, USA). The spectra were recorded with a resolution of 4 cm^−1^ (64 scan) in the range of 4000–650 cm^−1^. The Essential FTIR^®^ version 3.50.194 was used to further analyze the spectra.

#### 2.4.3. Wettability

Measurements of the contact angles exhibited by the layers composing the material CA/PG+GEN were made to evaluate the hydrophilicity characteristics of the scaffold layers, using a Drop Shape Analyzer (DSA30S, Krüss, Hamburg, Germany) through the application of 2 µL of deionized water. Each contact angle value corresponded to the mean value of the left and right contact angle at a given point in time.

#### 2.4.4. Thermal analyses

Thermogravimetric analysis was performed on analyzer Q-50 (TA Instrument Inc., Waters LLC, New Castle, DE, USA). Samples (~10 mg) were placed in alumina pans, and runs were carried out in the range 37–400 °C, with a heating rate of 10 °C/min under nitrogen atmosphere. The thermal behavior of the obtained scaffold was evaluated by differential scanning calorimetry (DSC). The samples were heated in the temperature range from 37 to 400 °C at a heating rate of 10 °C/min under nitrogen DSC Q2000 (TA Instrument Inc., Waters LLC, New Castle, DE, USA).

### 2.5. Antimicrobial Activity

The antibacterial activity of the scaffold was evaluated against *S*. *aureus* and *P*. *aeruginosa* by means of a modify Kirby–Bauer test [20]. Briefly, nutritive agar was inoculated with cellular solutions of bacteria with a concentration adjusted to 1 × 10^6^ CFU mL^−1^. Disc samples of the scaffold (10 mm diameter) were deposited onto the inoculated agar. As positive control, paper discs of same size were soaked with 100 µL of the gentamicin solution. As negative control, UV sterilized samples of the two-layer material without antibiotic were used. The plates were incubated at 37 °C for 24 h, after which the inhibition halos present in the cultures were measured. The obtained values are the mean ± standard deviation (SD) of the measurements carried out on samples analyzed three times.

### 2.6. Statistical Analysis

All the quantitative data were recorded as a mean ± SD. The statistical differences were determined with one-way ANOVA with post-hoc Tuckey. Probability of the data was considered statistically significant for *p*-values less than 0.05 and statistically highly significant for *p*-values less than 0.01. The results were marked with (*) for *p* < 0.05 and (**) for *p* < 0.01.

## 3. Results

### 3.1. Characterization of the PG and CA Scaffold

#### 3.1.1. Scanning Electron Microscopy—SEM

**Figure 1:**
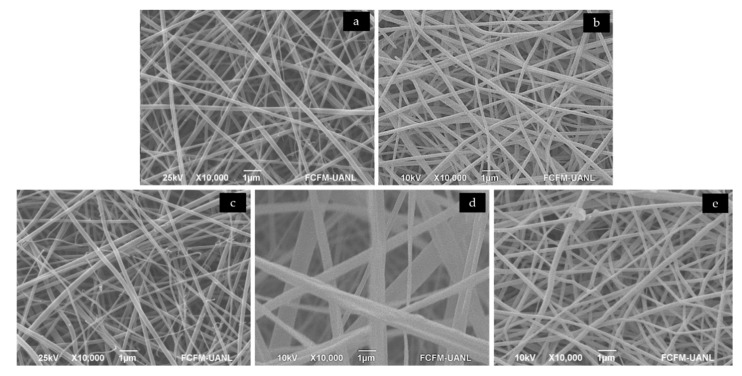
SEM images showing the fibers morphology spun from (**a**) polyvinyl pyrrolidone (PVP) and gelatin solution, (**b**) PVP and gelatin with gentamicin, (**c**) cellulose acetate solution, (**d**) cellulose acetate layer on top of polyvinylpyrrolidone:gelatin (PG)+gentamicin (gen) layer and (**e**) PG+gen layer on top of cellulose acetate layer.

Through SEM, the morphology of the fibers that constitute the polymeric scaffold was observed. Figure 1 shows the structure of the fibers that compose the different layers of the scaffold, which were processed and observed as comparison to evaluate the changes due to the addition of gentamicin in the PG layer or the ones that occurred in the fibers of CA due to its processing using the PG+GEN layer as a collector. All the fibers processed from the respective solution showed smooth edges without the presence of beads. All the samples of each layer presented random fiber patterns. The diameter distribution of the fibers observed is shown in Table 1.

#### 3.1.2. Attenuated Total Reflection Fourier Transform Infrared Spectroscopy—ATR-FTIR

**Figure 2:**
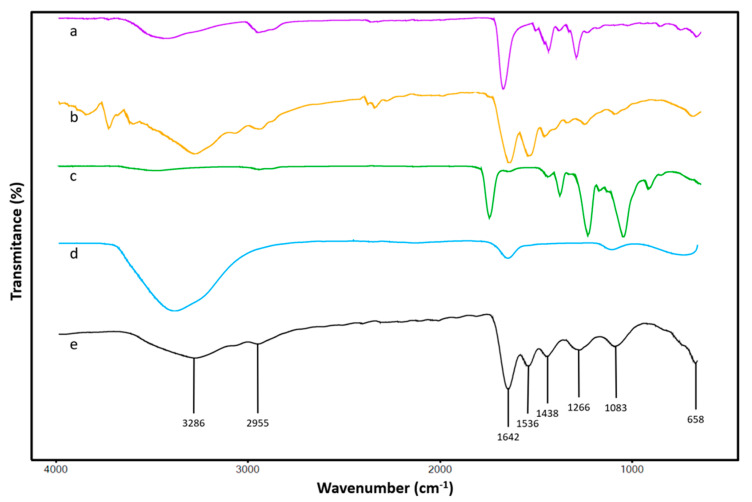
Attenuated total reflection Fourier transform infrared spectroscopy (ATR-FTIR) spectra from (**a**) PVP, (**b**) gelatin (GEL), (**c**) cellulose acetate (CA), (**d**) gentamicin and (**e**) scaffold. The latter shows the characteristic signals of all the components of this material.

Samples of the scaffold were taken to be analyzed by FT-IR to observe the changes in the chemical groups present in the fibers when compared with the used materials for the elaboration of the polymeric solutions before to be processed. Figure 2 shows the characteristic signals of all components present in the final scaffold.

#### 3.1.3. Wettability

**Figure 3:**
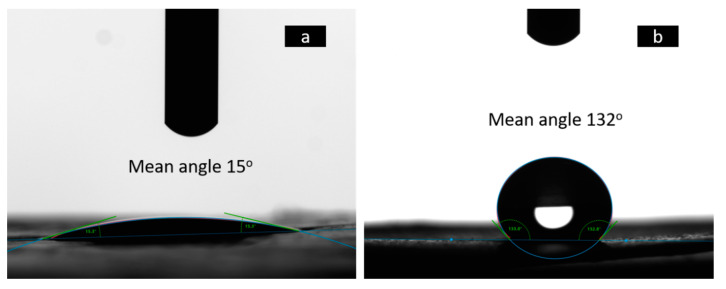
Determination of the wettability characteristics of the scaffold layers by measuring the value of their contact angle: (**a**) the first layer of PG+gen and (**b**) CA—the second layer.

**Figure 4:**
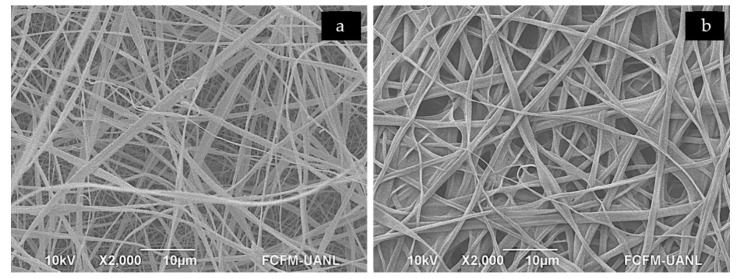
SEM images showing the morphology of the (**a**) scaffold CA layer fibers and the changes it suffered after (**b**) scaffold CA layer was exposed to water.

The wettability behavior of the layers surface was investigated by determining the values of their contact angles. Figure 3 presents the results of layers (a) PG+gen and (b) CA with mean values of 15.32° ± 0.34° and 132.88° ± 0.12°, respectively. SEM observations were performed to observe the morphological changes of the CA fibers after its exposure to water, as can be seen in Figure 4; they start to melt after exposure to water, which suggests that even the hydrophobic layer CA could be solubilized.

#### 3.1.4. Thermal Analyses

**Figure 5:**
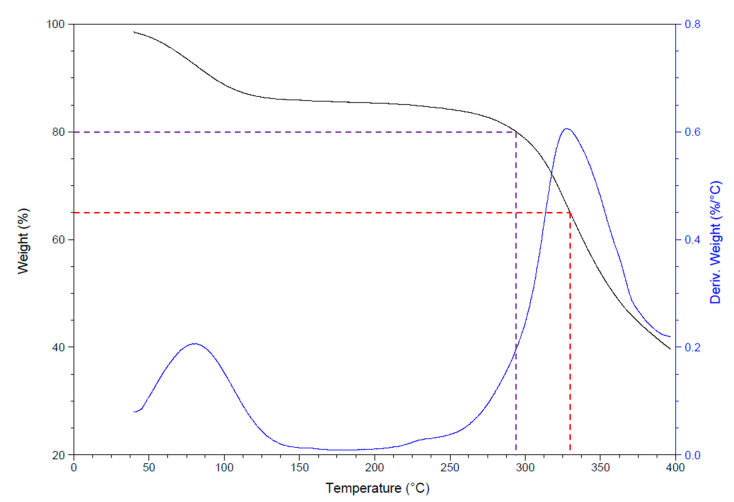
Thermogravimetry of the scaffold. The purple line indicates the loss of the first 20% of the sample mass (*T*_d20%_), and the red line indicates the temperature point for the half mass loss (*T*_d50%_).

The thermogravimetric (TGA) results of the scaffold are shown in Figure 5. The *T*_d20%_ of the material was in a range of 294.3 ± 1.7 °C, while the T_d50%_ was in the range of 359.6 ± 1.1 °C.

**Figure 6:**
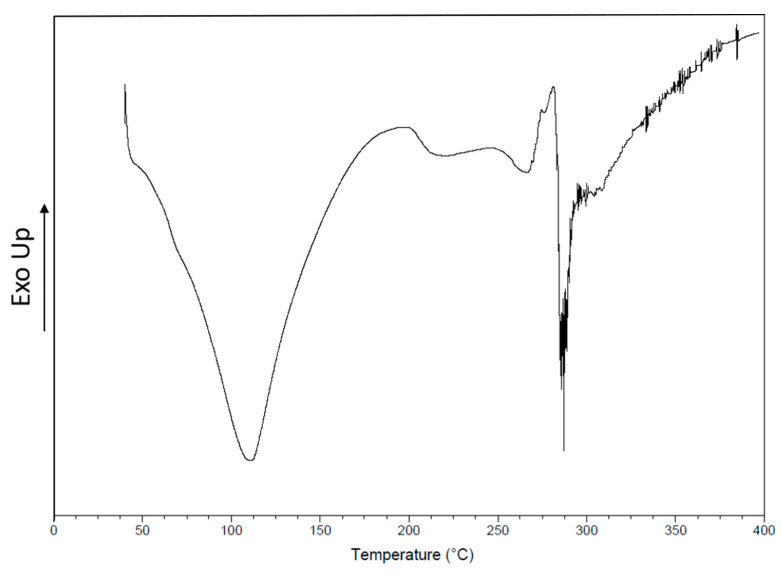
Differential scan calorimetry (DSC) curve showing the thermal behavior of the scaffold during a first heating run.

Representative DSC thermogram of the prepared material is shown in Figure 6. A broad endothermic peak between initial temperature (37 °C) and 170 °C with a peak maximum at 120 °С.

### 3.2. Antimicrobial Activity

**Figure 7:**
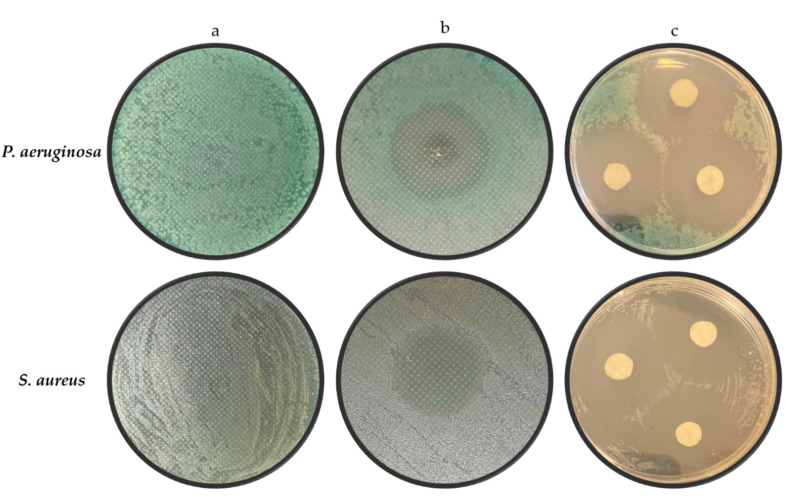
Inhibition halos observed in the bacteria cultures due to the presence of the different treatments: (**a**) negative control using scaffold samples without gentamicin, (**b**) cultures incubated with scaffold sample and (**c**) positive control with gentamicin disks.

Growth inhibition halos due to gentamicin action onto the cultures of both bacteria were observed (Figure 7). The mean values in millimeters of the inhibition zone generated by the presence of the two-layer material without antibiotic (negative control), samples of the scaffold, and the discs with gentamicin solution (positive control) are shown in Table 2.

## 4. Discussion

The diameter of the fibers observed in the PG layer resulted in an average of 56 ± 16 nm; when compared with these fibers, the ones from the PG+gen layer of the scaffold showed a considerable increased diameter. This could be attributed to the addition of the gentamicin in the formulation [21]; nonetheless, thinner fibers have been obtained in papers with similar compositions [7]. Analyzing the distribution of the CA fibers, very significant statistical difference can be observed, considering these fibers as submicrometric structures. Comparing the CA fibers of this study with ones obtained in other essays with similar composition, the mean diameter of the fibers of this project are smaller [22,23]. The fibers resulting from processing CA as a second layer for the scaffold increased considerably, showing statistical difference. This was expected, as the PG+gen layer could act as an insulator on the collector, affecting the process of the formation of the second layer fibers [21].

Something similar occurred when analyzing the pore diameters exhibited in the scaffold; it was observed that there is statistical difference between the pores of the CA layer with those of PG and PG+gen (Table 1). Nonetheless, the pores from the CA layer are still smaller than the average size of the bacterial cells, meaning that the CA layer could be considered as a physical barrier against the colonization by bacterial microorganisms once the scaffold is placed over the wound [24].

The ATR-FTIR analysis of the scaffold allowed one to observe most of the characteristic signals from components of the material. Due to PVP, peak in 1438 cm^−1^ corresponds to in-plane bending vibration from C–H [25] and signal at 1642 cm^−1^ corresponds to C=C vibrational stretching of the amide I, which is the most characteristic group of this polymer [26]. Taking CA (Figure 2c) into consideration, is important to mention that the band at 900 cm^−1^ assigned to C–O–C stretching at β-(1→4)-glycosidic linkages, which is designated as an “amorphous” absorption band, increased in intensity and occurred in amorphous samples, but in the case of the fibers forming the scaffold obtained in this work, this signal disappeared, suggesting that the amorphous configuration of the CA is reduced in the material [27]. The peak 1266 cm^−1^ corresponded to the C–C symmetric stretching. The peak at 1370 cm^−1^, related to the angular distortion of the CH in the ester methyl group, and the one at 1730 cm^−1^ can be assigned to the acetyl group on the polymeric chain [28]; they were not detectable in the scaffold specter. Again, the presence of amorphous cellulosic samples can be confirmed by the shift of the band from 900 cm^−1^ [27], corresponding to the C–H stretching vibration, the higher wave number values and the strong decrease in the intensity of this band. In the case of the fibers obtained during this work, the band shifts to 2955 cm^−1^, which could mean that amorphous CA presence in the fibers is at a minimum. The typical porcine GEL presents three important regions, and the material obtained in this work presented them all. First is the band at 1536 cm^−1^ that is caused by deformation of the N–H bonds in the amide II; the band at 1642 cm^−1^, which is the same that the one in PVP; and the band at 3286 cm^−1^ that corresponds to N–H stretching mode of hydrogen bonded amide groups [16,29]. For the gentamicin, the band at 658 cm^−1^ was confirmed given its importance as a signal for the determination of this compound, as well as two more bands at 1536 and 1642 cm^−1^ related to the primary aromatic amines [17]; finally, a reported signal at the region around 3286 cm^−1^ showed characteristic for the stretches of the N–H amino groups of the antibiotic [30]. It has been reported that the enrichment of polymer scaffolds with pro-adhesive sequences enhances the biocompatibility of the biomaterial [9]. Regarding the material obtained in this work, one of the mainly functional groups contained is the amino, which could promote cell adhesion and proliferation, thus improving the regeneration of some tissues [31].

Regarding the wettability of the scaffold layers, the results for the PG layer were as expected due to the known characteristics of the polymers but were contrasting for the CA layer [8]. The resulting contact angle for the PG+gen layer suggests it could be considered as a hydrophilic material. In the case of the CA layer, the resulting contact angle suggests hydrophobic characteristics [32]. The scaffold physical properties could assist in cell migration and differentiation [33], according to previously reported results where the lower the contact angle of the material, the greater the hydrophilicity and the higher the biocompatibility [34]. Considering these results, the potential application of this material as a dressing for wounds exists, given that the layer that would be in direct contact with the tissue would be the PG+gen and the CA layer could fulfill the role as physical barrier to avoid colonization by microorganisms [35].

The thermal behavior of the scaffold was as expected according to the results of some studies done before. LaFountaine et al. [36] reported degradation temperatures for PVP around 175 °C, and in the case of the CA some articles had shown that the degradation temperature is around 225 °C [12]; nonetheless, the scaffold obtained in this work only lost around 20% of its weight over 100 °C above this temperature. This result could be attributed to the interactions among the polymers that constitute the scaffold, considering that the presence of GEL in the scaffold could be increasing the degradation temperature. GEL thermal degradation occurs in two stages, the first one from room temperature to 200 °C, losing around the 12% of weight [37], phenomenon that could be present in the results of this paper as seen in Figure 5. The next stage for GEL degradation occurs between 200 and 400 °C [37,38,39], similar to results observed in this study.

The DSC analysis starts with an endothermic event attributed to desorption of water from the polymers that constituted the scaffold [12] and related with the first denaturation of the GEL [40]. Endothermic transition corresponding to *T*_g_ at 195 °С and endothermic peak at 265 °С corresponding to *T*_m_ of the scaffold were observed; these results could be attributed to the presence of CA and GEL [38,40].

The results from TGA and DSC suggest that the presence of GEL in the scaffold improves the thermal stability of PVP and CA.

The antibacterial activity of the mat was evaluated against *S*. *aureus* and *P*. *aeruginosa* by means of the inhibition Kirby-Bauer test (Figure 7). The halos resulting from the scaffold test present no statistical difference with the gentamicin solution. These results, being compared with others reported previously in different articles [41,42], suggest that the release of gentamicin from polymeric fibers allows for its correct activity against bacteria, preventing the growth of these microorganisms, and confirming that the drug did not suffer damage due to the electrospinning process.

## 5. Conclusions

A two-layer scaffold was obtained by electrospinning, consisting of one layer of PVP-GEL and one layer of CA, whose antibacterial activity is comparable to that observed in a solution with gentamicin but with a smaller amount of the antibiotic needed to prevent the bacterial growth of *P*. *aeruginosa* and *S*. *aureus*.

Considering the physicochemical and microbiological properties of this scaffold, there is the possible application of this material as a wound dressing, because the layer that would be in direct contact with the tissue would be the hydrophilic PG+gen and together with the stability of the antimicrobial activity presented after the electrospinning process would allow greater contact of the antimicrobial agent with the wound; on the other hand, the hydrophobic CA layer would play the role of a physical barrier to prevent colonization by microorganisms, until solubilization.

## Figures and Tables

**Table 1:** Average diameter of fibers and pores.

Structure	PG	PG+gen	CA	Scaffold
Fibers	56 ± 16 *	124 ± 26	138 ± 66	307 ± 110 *
Pores	127 ± 47 *	372 ± 146	309 ± 135	472 ± 209 *

The units of all data are nanometers (nm). * means statistical difference with *p* = 0.01.

**Table 2:** Antimicrobial activity obtained for controls and scaffold.

Bacteria	a	b	c
*P. aeruginosa*	0	24.82 ± 1.10	25.17 ± 1.46
*S. aureus*	0	25.62 ± 0.86	25.72 ± 0.99

Inhibition zones after incubating the bacteria: (**a**) negative control, (**b**) scaffold samples and (**c**) positive control. All measurements are in millimeters (mm).
